# Does Small Ruminant Lentivirus Infection in Goats Predispose to Bacterial Infection of the Mammary Gland? A Preliminary Study

**DOI:** 10.3390/ani11071851

**Published:** 2021-06-22

**Authors:** Daria Urbańska, Ryszard Puchała, Justyna Jarczak, Michał Czopowicz, Jarosław Kaba, Karina Horbańczuk, Emilia Bagnicka

**Affiliations:** 1 Institute of Genetics and Animal Biotechnology, Polish Academy of Sciences, Postepu 36A, 05-552 Jastrzębiec, Poland; daria.m.urbanska@gmail.com (D.U.); k.horbanczuk@igbzpan.pl (K.H.); 2 Applied Physiology Unit, Military Institute of Hygiene and Epidemiology, Kozielska 4, 01-001 Warsaw, Poland; rapuchala@gmail.com; 3 Biobank Lab, Department of Molecular Biophysics, University of Łódź, Pomorska 139, 90-235 Łódź, Poland; justyna.jarczak@biol.uni.lodz.pl; 4 Division of Veterinary Epidemiology and Economics, Institute of Veterinary Medicine, Warsaw University of Life Sciences-SGGW, Nowoursynowska 159c, 02-776 Warsaw, Poland; michal_czopowicz@sggw.edu.pl (M.C.); jaroslaw_kaba@sggw.edu.pl (J.K.)

**Keywords:** caprine arthritis-encephalitis, mastitis, parity, pathogenic bacteria, polish white improved, polish fawn improved, small ruminants, udder

## Abstract

**Simple Summary:**

The health and functionality of the mammary gland are important factors in animal welfare and milk production. Inflammation of the udder is associated with reduced milk yield and dairy product quality. Even though mastitis is usually caused by bacterial, fungal, or algae infections, some studies have suggested that infection with small ruminant lentivirus (SRLV), causing caprine arthritis-encephalitis (CAE), can also cause mastitis in small ruminants. Its pathophysiology is not, however, fully understood. Therefore, the aim of this study was to determine whether seropositive goats were more susceptible to bacterial infections of the udder than uninfected goats. A higher prevalence of pathogenic bacteria was identified only in seropositive goats in the 5th or further lactation. This indicates that a relationship may exist between a long-lasting SRLV infection and decreased resistance of the udder to bacterial infections, even though the seropositive goats enrolled in this study had no clinical signs of CAE.

**Abstract:**

The aim of this study was to determine whether asymptomatic small ruminant lentivirus seropositive (SRLV-SP) goats were more susceptible to bacterial infection of the udder when lactating by comparing the presence and species of pathogenic bacteria in their milk with the values for seronegative goats (SRLV-SN). Milk samples were collected during morning milking on days 20, 40, 60, 150, and 210 of lactation for three consecutive years and subjected to bacteriological examination. *Staphylococcus caprae* and *S. xylosus* were the most frequent strains identified in both SRLV-SP and SRLV-SN goats. The prevalence of pathogenic bacteria was the highest in the 1st lactation, regardless of SRLV status. Moreover, the prevalence of pathogenic bacteria was significantly higher in SRLV-SP goats, but only those in the 5th or further lactation (*p* = 0.010). This suggests a relationship between long-lasting SRLV infection and susceptibility to bacterial infections of the udder.

## 1. Introduction

The health and functionality of the mammary gland are important factors in the welfare of dairy animals and milk production, as well as in dairy product quality and profitability for farmers and the dairy industry. Mastitis is typically caused by bacterial, fungal, or algal infections, and usually manifests as an increase of milk somatic cell count (SCC) [1]; however, pathogenic bacteria, including *Staphylococcus aureus*, have been detected in about 20% of goat milk samples with SCC < 1 × 10^6^/mL [2].

*S. aureus*, a member of the coagulase-positive staphylococci (CPS), often causes mastitis in dairy cattle, and its presence is usually associated with increased SCC. However, some researchers indicate that *S. aureus* is more likely to cause subclinical than clinical mastitis in cows [3]. Even if so, no effective vaccine against mastitis caused by *S. aureus* has so far been found [4,5]. In small ruminants, *S.* *aureus* is only sporadically present in clinical mastitis [6]; however, the infection often takes a very severe course, even leading to gangrene [7]. Moreover, the presence of *S. aureus* in milk, and particularly its thermostable toxins, which are resistant to pasteurization, can also pose a threat to human health by causing food poisoning [8]. Another coagulase-producing staphylococcus—*S. intermedius*—has also been isolated from goat milk [2].

Various species of coagulase-negative staphylococci (CNS), such as *S. epidermidis*, *S. xylosus*, *S. chromogenes*, and *S. simulans*, as well as various streptococci, enterobacteria, arcanobacteria, corynebacteria, pasteurellae, enterococci, and *Pseudomonas* spp. have been isolated from goat and sheep milk or mammary gland tissues [1,2,6]. Although the CNS were considered a minor and environmental, opportunistic bacteria, it has recently become evident that they may also cause subclinical mastitis in goats [4,5,9,10]. Furthermore, *Escherichia coli*, *Listeria monocytogenes*, and *Salmonella* spp. may also cause mastitis in dairy goats [10]. Spuria et al. [1] suggest that infection with small ruminant lentivirus (SRLV) causes mastitis in small ruminants. However, it is not known whether this inflammation is caused by the virus itself or the presence of SRLV in the udder reduces its resistance to the secondary bacterial infection.

SRLV is a single-stranded RNA virus belonging to the Lentivirus genus of the *Retroviridae* family, together with immunodeficiency viruses of humans (HIV), cats (FIV), and cattle (BIV). SRLV infects monocytes, macrophages, and dendritic cells but, unlike immunodeficiency lentiviruses, it does not infect lymphocytes. SRLV causes caprine arthritis encephalitis (CAE) in goats and maedi-visna disease (MV) in sheep. Thus far, five SRLV genotypes with several subtypes have been described: A (22 subtypes), B (five subtypes), C, D, E (two subtypes) [11]. The most common are genotypes A and B. In Poland A1, A5, A12, A13, A16, and A17, and B1 and B2 have been detected [12,13,14].

SRLV is vertically transmitted to the offspring through the lactogenic route, i.e., infected macrophages and epithelial cells present in milk or can be transmitted horizontally via long-lasting contact with infected animals (infection via respiratory or sexual routes) [15,16]. Interspecies transmission between sheep and goats can also occur [17,18]. SRLV infection is lifelong, with an incubation period lasting many months or even years. The most characteristic clinical signs are arthritis in goats and pneumonia in sheep, while mastitis can occur both in sheep and in goats [17,19].

Contradictory results are reported in the literature in relation to the effect of SRLV infection on the immune system. Some authors have reported differences in the expression of several cytokines during SRLV infection, both in blood leukocytes (BL) or in blood serum and milk somatic cells (MSC) [20,21], implying that the virus probably suppresses the immune response of the host by preventing the activation of defense mechanisms. The independent immune response of the mammary gland initiated in response to the presence of SRLV consists of a specific pattern of gene expression, the mammary gland being one of the target organs of SRLV. In contrast, Reczyńska et al. [22] reported no differences between SRLV-seropositive (SRLV-SP) and seronegative (SRLV-SN) goats regarding the expression profile of cathelicidins (antimicrobial peptides) in BL and MSC; however, they also observed higher concentrations of only one positive acute phase protein (APP) produced in response to inflammation in blood leukocytes, namely serum amyloid A (SAA), and a lower concentration of SAA and ceruloplasmin (Cp) in milk. The authors hypothesized that this lack of observed differences in the concentrations of other APPs indicated capability of SRLV to inhibit both systemic and local immune responses. This could occur by suppression of the host defense mechanism against the viral infection or it was possible that the immune response of the host remained in a neutral, non-inductive state. However, similarly to Jarczak et al. [21], they stressed that the decreased SAA and ceruloplasmin (Cp) concentrations found in the milk of SRLV-SP goats could indicate that the mammary gland was coping with the virus; SAA could stimulate the differentiation of monocytes to macrophages, which was essential for viral multiplication, while the main function of Cp was to remove reactive oxygen species, a deadly weapons against pathogens [23].

It appears that elevated expression of some pro-inflammatory cytokines only activates the production of SAA in blood serum; reduced concentrations of SAA and Cp have been observed in milk during SRLV infection in goats. If SRLV disturbs the immune status of the mammary gland, the mammary gland may become more susceptible to bacterial infections. However, it is still not clear whether SRLV impairs the local immune system in the mammary gland. Despite this, some studies have indicated a correlation between maedi-visna diseases and subclinical mastitis in sheep. Asadpour et al. [24] reported that more than 15% of sheep with mastitis were infected with SRLV, and Houwers et al. [25] noted that more than 60% of SRLV-infected ewes had mastitis. In addition, Ryan et al. [26] and Smith and Cutlip [27] suggested that SRLV-SP goats could be more susceptible to bacterial infection of the mammary gland, especially infection caused by non-hemolytic staphylococci. Tariba et al. [28] also reported a similar relationship between infection and the occurrence of mastitis. However, Nord [29] indicated that the prevalence of bacterial infection of the udder was similar between SRLV-SP and SRLV-SN goats.

The aim of this study was to determine whether asymptomatic SRLV infection predisposed lactating goats to bacterial infection of the udder.

## 2. Materials

The study was conducted in the years 2014–2016 in a herd of 50 dairy goats located in central Poland. Blood had been regularly taken from all animals for serological testing to diagnose SRLV infection for 20 years before the study. Three enzyme-linked immunosorbent assay (ELISA) kits had been used: ELISA Checkit CAEV/MVV (Dr. Bommeli AG, Bern, Switzerland) in the period 2001–2007—based on whole virus antigen from the genotype A SRLV strain; Pourquier ELISA Maedi-Visna/CAEV Serum Verification (Institut Pourquier, Montpellier, France) in the period 2008–2012—based on recombinant gp46 (transmembrane)/p28 (capsid) antigen; ID Screen MVV-CAEV Indirect Screening test (ID.vet Innovative Diagnostics, Grabels, France) since 2013—based on whole virus antigen from the genotype B SRLV strain. The presence of the virus in the herd was also confirmed by isolation [30], with genotype A being the most prevalent [14]. Co-infections (subtypes A12/B2 and A1/B1/A12) were also found.

A total of 40 goats, i.e., 21 Polish White Improved (PWI) and 19 Polish Fawn Improved (PFI) goats, were included in the study. Their age at enrollment ranged from 1 to 8 years, with the median (IQR) of 3 (2–5) years. Nine goats (22.5%) participated in the study for three lactations, 11 goats (27.5%) for two lactations, and 20 goats (50.0%) for only one lactation.

In addition to ELISA, the goats were tested for the presence of the virus by RT-qPCR according to Brinkhof et al. [31]; however, the viral load was below the level of detection. Twenty-four goats (60.0%) were SRLV-SP for the whole study, 11 goats (27.5%) were SRLV-SN for the entire study, and five goats (12.5%) seroconverted during the study.

The goats were placed in group pens and fed according to INRA recommendations adapted to Polish conditions [32]; they had free access to water, and they were milked twice a day in a parallel milking parlor. The SRLV-SN goats were milked first to avoid the virus being spread through milking cups.

In total, 235 milk samples were collected—80 (34.0%) from SRLV-SN goats and 155 (66.0%) from SRLV-SP goats. Thirty milk samples (12.8%) were collected in the 1st lactation, 20 (8.5%) in the 2nd, 38 (16.2%) in the 3rd, 56 (23.8%) in the 4th, and 91 (38.7%) in the ≥5th lactation (Table 1).

## 3. Methods

### 3.1. Bacteriological Examination

Foremilk samples were collected in a sterile manner during morning milking on days 20, 40, 60, 150, and 210 of lactation, over three consecutive years (2014–2016), and analyzed for the presence of bacteria using standard bacteriological methods. Briefly, 100 µL of each milk sample were inoculated on BD Columbia CNA agar with 5% sheep blood to culture Gram-positive bacteria and MacConkey agar to culture Gram-negative bacteria (BioMérieux, Craponne, France). The plates were incubated at 37 °C for 24–48 h. Bacterial infection was defined as the presence of a bacterial colony on the plate with the milk sample. The individual species of bacteria were identified using the Vitek2 system (BioMérieux, Carponne, France), which can identify a wide range of microorganisms by fluorescence-based technology.

### 3.2. Statistical Analysis

Numerical data were expressed as the median, interquartile range (IQR) and range. Categorical data were presented as counts and percentages and compared between groups using Pearson’s χ^2^ test. The 95% confidence intervals (CI 95%) for proportions were calculated using the Wilson score method. The relationship between SRLV status and prevalence of bacteria in milk samples was investigated using the generalized binary logistic mixed model. The variable *Goat* was forced into the model and fitted as a random effect (*G*) to control for the dependence of multiple observations coming from a single goat. Four variables were fitted as fixed effects: the main explanatory variable corresponding to the change of SRLV status in time (X_SRLV_ith parity_), which described the SRLV-specific serological status of a goat in a given parity class (lactation) with seronegative status, regardless of lactation, being a baseline category; and three potential confounding factors: breed (X_PWI_) as a nominal dichotomous variable with PWI being a baseline category; parity as a 5-class ordinal variable with the 1st lactation being the baseline category and all lactations ≥5th included in a single category (X_L2_ through X_L5_); milking number (stage of lactation) as ordinal variable with the 1st milking being the baseline category (X_M2_ through X_M5_). The three potential confounding factors were eliminated from the model according to the backward stepwise procedure unless significant. The model was expressed by the following formula:

(1) $$P\left(Y=1\right)=\frac{1}{1+e^{-\left(B_{0}+B_{\mathrm{PFI}}\times X_{\mathrm{PFI}}+B_{L\_n}\times X_{L\_n}+B_{M\_n}\times X_{M\_n}+B_{\mathrm{SRLV}\_\mathrm{ith}\_\mathrm{parity}\;}\times X_{\mathrm{SRLV}\_\mathrm{ith}\_\mathrm{parity}}+G\right)}}$$

where P(Y = 1) was the probability of isolating bacteria from a milk sample, B_0_ was the intercept, and B with a subscript were regression coefficients for breed (B_PWI_), subsequent lactations (B_L_n_), subsequent milking (B_M_n_), and serological status in subsequent lactations (B_SRLV_ith_parity_).

Strength of relationship between factors and prevalence of bacteria was expressed using odds ratio (OR). All statistical tests were two-tailed. The significance level (α) was set at 0.05 and the Bonferroni correction was used for multiple comparisons. Univariable statistical analysis was performed, and the graphs were prepared in TIBCO Statistica 13.3 (TIBCO Software Inc., Palo Alto, CA, USA). Mixed model was developed in IBM SPSS Statistics 26 (IBM Corporation, Armonk, NY, USA).

## 4. Results

Bacteria were detected in milk samples collected from 31 of 40 goats enrolled in the study (77.5%; CI 95%: 62.5%, 87.7%). Of the 235 milk samples, 95 were positive (40.4%). Five of them (5.3%) contained two different species of bacteria; in all cases, they were two different staphylococci (*S. gallinarum* and *S. lentus*, *S. epidermidis* and *S. xylosus*, *S. lugdunennis* and *S. caprae*, *S. lugdunennis* and *S. gallinarum*, *S. epidermidis* and *S. scuri*). The identified species of bacteria in goat milk are shown in Table 2. Staphylococci were present in 90 of the 95 samples containing bacteria (94.7%). The prevalence of bacteria was 45.8% (CI 95%: 38.2%, 53.7%) in milk samples from SRLV-SP goats and 30.0% (CI 95%: 21.1%, 40.8%) in milk samples from SRLV-SN goats. Among the identified bacteria, the most common species were *S. caprae* and *S. xylosus*, followed by *S. gallinarum*.

**Figure 1:**
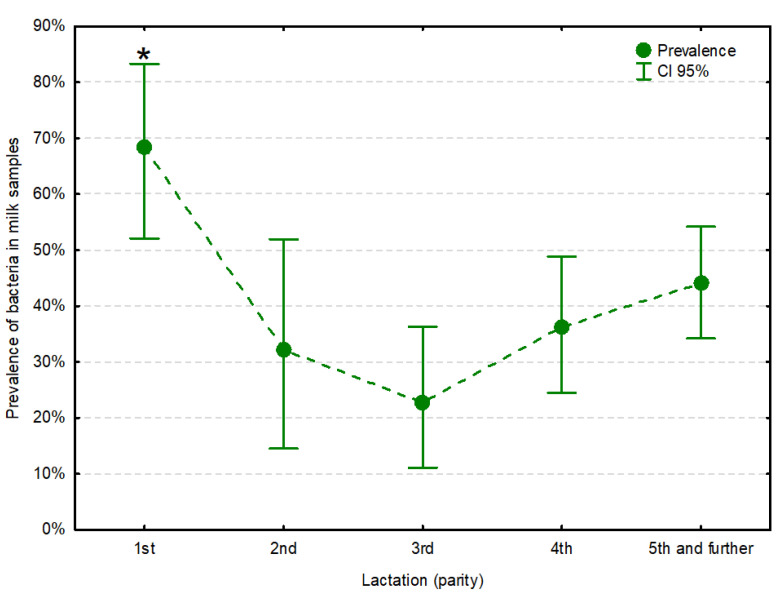
Prevalence of bacteria in milk samples from subsequent lactations, regardless of small ruminant lentivirus infection status. The asterisk (*) indicates statistical significance at α = 0.05.

No difference in the prevalence of bacteria was observed between milk samples collected from different breeds, regardless of the SRLV status of the animals (F_1220_ = 0.01, *p* = 0.909). Neither was the stage of lactation significantly linked to the prevalence of bacteria (F_4221_ = 2.13, *p* = 0.079), even though the highest prevalence of bacteria was found on the 150th day of lactation, i.e., in full lactation. Hence, these two potential confounders were eliminated from the model as insignificant. The prevalence of bacteria was significantly linked to the parity (F_4225_ = 2.85, *p* = 0.025). It was the highest in milk samples from the first lactation, and significantly lower in samples from subsequent lactations (Figure 1, Table 3).

**Figure 2:**
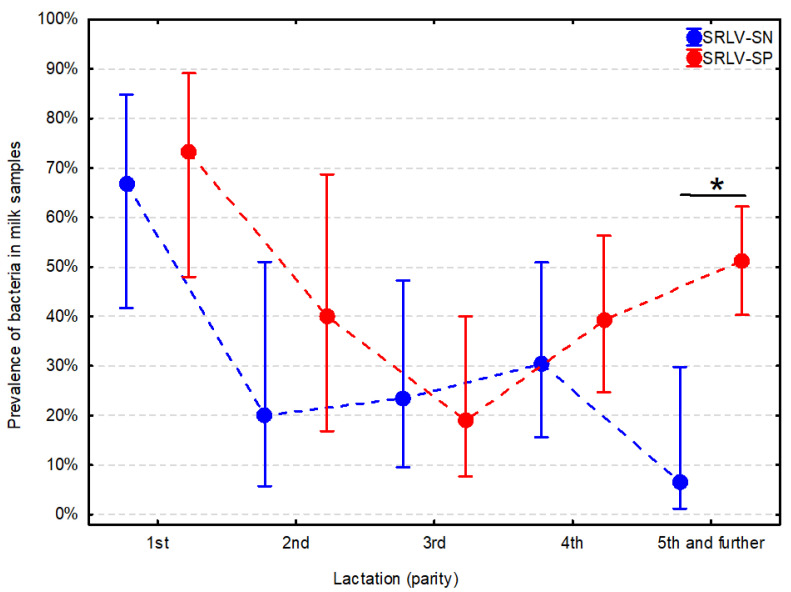
Prevalence of bacteria in milk samples from small ruminant lentivirus-seropositive (SRLV-SP) and seronegative (SRLV-SN) goats in subsequent lactations. Whiskers indicate CI 95% and the asterisk (*) indicates statistical significance at α = 0.05.

Controlling for potential confounding factors, the significant relationship between the prevalence of bacteria and SRLV status was present; however, it was significantly modified by parity (Table 3). In the SRLV-SP goats, the prevalence of bacteria was significantly higher only in the ≥5th lactation (*p* = 0.012) (Figure 2, Table S1). The SRLV-SP goat in the ≥5th lactation was roughly 13-fold more likely to have bacteria in milk than the SRLV-SN goat (Table 3).

## 5. Discussion

The goat breed appeared to have no influence on the prevalence of bacteria, regardless of the SRLV status of the animals. In fact, transcriptomic analysis revealed no significant differences between these two breeds regarding gene expression (~50 K), except for that of the *Capra hircus agouti* signaling protein (ASIP), responsible for the coat color [29]. Thus, our result indicates that there are no genetic differences in the susceptibility to mastitis between those two Polish dairy goat breeds. Even though in a study of the entire Polish active goat population Bagnicka et al. [33] indicated a higher SCC in the milk of the PFI than in that of the PWI goats but not in lactose content, which is also an indicator of mastitis. On the other hand, it is known that SCC in goat milk depends on many factors, not only on mammary gland infection and the presence of pathogens.

In contrast to our present findings, Nowicka et al. [34] found no differences in the prevalence of bacteria between SRLV-SN and SRLV-SP goats, with bacteria being observed in 53% of tested samples: 59% for SRLV-SP and 51% for SRLV-SN. In their study, the CNS were also the most commonly found bacteria in milk (92% of samples) of both SRLV-SP and SRLV-SN goats.

Some bacterial species such as *S. caprae*, *S. xylosus*, or *S. chromogenes*, were detected in milk samples from both the SRLV-SN and SRLV-SP goats; however, their prevalence slightly differed between groups. In contrast, *S. simulans, S. arlettae, S. vitulinus,* and *S. carnosus* were only isolated in the milk of the SRLV-SP goats, and *S. capitis* only in SRLV-SN goats. Deinhofer and Pernthaner [10] identified bacteria in only 18% of 2243 milk samples of SRLV-free dairy goats (Togenburger, German White and Colored Improved, and cross breeds); most of the bacteria belonged to the *Micrococcaceae* (89%) genus with *Pasteurella* spp., *E. coli*, and *Actinomyces* spp. being observed in the remaining samples, which is at odds with our results because the prevalence of bacteria in SRLV-SN groups in the present study was as high as 30%. However, in both studies, the most prevalent bacteria were staphylococci.

Other studies have found *S. simulans* [20] and *S. caprae* to be the most common pathogens [5], the latter colonizing the skin and the mammary gland of goats. Contreras et al. [35] reported the presence of bacteria in 40% of 538 goat milk samples from Alpine, Saanen, and Nubian breeds; however, their SRLV infection status was not investigated. Almost all bacterial strains (>95%) belonged to the *Staphylococcus* genus (CNS or CPS), with *S. epidermidis* being the most common (67% of samples). In addition, examples of *Streptococcus* spp., *Corynebacterium* spp., *Enterobacteriaceae*, and *Bacillus* spp. were found. Of these species, only *S. epidermidis* was found in the present study. Boscos et al. [36] observed the presence of bacteria in 29% of 186 samples infected mainly with CNS (62%). The other identified bacteria were *S. aureus* (19%) and *Streptococcus* spp. (9%). While CNS was also the most common group of bacteria found in our study, the *S. aureus* was found much less often, and no streptococci were found in any group at all.

Peterson et al. [37] proposed that *S. warneri* and *S. saprophyticus* were likely to cause udder infection; these species were more frequently observed in milk from SRLV-SP animals. In turn, Deinhofer and Pernthaner [10] reported that *Streptococcus epidermidis*, *S. simulans*, *S. lugdunensis*, *S. chromogenes*, and *S. warneri* infections were connected with higher SCC levels and pathological changes in udder tissues.

Other authors have also reported a much higher prevalence of CNS in goat herds, ranging from 45% to 96% of milk samples, compared to 4% to 18% of milk samples for *S. aureus* [38], which is consistent with our findings. However, they also stressed that *S. aureus* was usually considered to have greater pathogenicity than CNS. This group of bacteria is predominant not only in goat milk but also in the milk of other dairy species [39]. Furthermore, some CNS such as S. *hemolitycus*, *S. epidermidis*, *S. chromogenes*, *S. warnei*, or *S. xylosus* are able to produce endotoxins such as pathogenic *S. aureus* [40], and of them, only *S. hemoliticus* was not found in our study. In contrast to *S. aureus*, it is believed that CNS causes rather mild inflammation in bovines and that the infection is self-curable, without antibiotic treatments [39,41].

As mentioned above, the most commonly observed form of SRLV in the studied herd was genotype A; this genotype used to be considered an ovine form of the virus [12], and it is possible that this genotype may cause milder lesions in goats. Indeed, Deubelbeiss et al. [42] found SRLV A4 viruses to be of low pathogenicity for goats; the studied goats had no signs of CAE, did not lame, had no neurological symptoms, and their carpal/metacarpal ratios did not deviate from the norm. No pathological lesions were observed during necropsy of the lung, synovial membranes of the joints, and the choroid plexus. However, all goats demonstrated some changes in the mammary gland, ranging from very mild (grade 1) in one goat, through more pronounced forms (grade 3) in three goats, to very strong (grade 4) in one. Clearly, this issue merits further investigation, especially because some relationship between maedi-visna disease and mastitis has been found in sheep [22,23]. In addition, it has been suggested that SRLV-SP goats are more susceptible to bacterial infection of the mammary gland, especially to non-hemolytic staphylococci [24,25,26]. However, contradictory results have also been presented; for example, similar frequencies of udder bacterial infections were in seropositive and seronegative animals [27]. A study of 140 udder halves infected with different strains of staphylococci [43] also concluded that new intra-mammary infections, as well as persistent ones occurring during the dry period, were not associated with SRLV status. Some results have suggested a positive relationship between parity and risk of subclinical mastitis [44,45,46]. Moreover, the higher prevalence of subclinical infections at the later stages of lactation was explained by infection persistence [44]. However, the SRLV serological status was not investigated in those studies.

The mammary gland is one of the organs targeted by SRLV infection. Reczyńska et al. [22] and Jarczak et al. [21] propose that the gland demonstrates a different local immune response to infection than the whole organism; their findings indicate different patterns of expression of APPs and cytokine genes, at both the mRNA and protein levels, in MSC compared to BL. They suggest that the infected udder may be combating the virus with no systemic response; however, if SRLV-positive animals are in good condition, without signs of CAE, their homeostasis may remain undisturbed. This observation appears to corroborate those of a study on the metabolomic profile of asymptomatic SRLV-SP Saanen done in Poland: of 130 studied metabolites, only two were influenced by SRLV infection. The authors concluded that the metabolism of asymptomatic goats was not substantially affected by SRLV [47].

The main limitation of this study is the low number of animals used and some milk samples missing at various stages of lactation.

Concluding, the prevalence of pathogenic bacteria appeared to be associated with SRLV infection. This may mean that, despite the lack of apparent signs of CAE, the homeostasis of the udder of infected goats may be disturbed by the virus, making them more susceptible to bacterial infection. Unfortunately, few comparable studies have been performed in this area, which precludes drawing more general conclusions. To obtain more comparable results, future studies regarding the influence of SRLV infection on the health of goats should include the age of the animals, the duration of the infection, the virus load, and, if possible, its genotype, as well as the presence, or lack, of any signs of CAE.

## 6. Conclusions

The difference in the prevalence of pathogenic bacteria between milk samples from SRLV-SN and SRLV-SP goats was observed only in the 5th and further lactations. This may indicate that long-lasting SRLV infection is related to the decreased resistance of the udder to bacterial infections, even if SRLV-infected goats remain asymptomatic.

## Figures and Tables

**Table 1:** Distribution of the milk samples according to parity and day of sampling.

Milking Day	Lactation Number (Parity)									
	1st	2nd	3rd	4th	5th	6th	7th	8th	9th	Total
20	6	4	8	15	10	8	2	0	0	53
40	6	5	9	13	9	5	1	0	0	48
60	6	3	9	9	7	7	2	0	1	44
150	6	5	7	11	9	6	2	0	1	47
210	6	3	5	8	9	8	2	1	1	43
Total	30	20	38	56	44	34	9	1	3	235

**Table 2:** Species of bacteria causing mastitis in 40 goats and their prevalence in milk samples from small ruminant lentivirus-seropositive (SRLV-SP) and seronegative (SRLV-SN) goats.

Pathogen	Type	Species	The Number (%) of Goats in Which the Bacterium Was Isolated	The Number (%) of Milk Samples From					
				The Total Study Population (*n* = 95)		SRLV-SP (*n* = 71)		SRLV-SN (*n* = 24)	
				*n*	%	*n*	%	*n*	%
Major	CPS	*S. aureus*	2 (5.0)	2	2.8	1	1.4	1	4.2
Minor	CNS	*S. caprae*	12 (30.0)	20	28.2	14	19.7	6	25.0
		*S. xylosus*	10 (25.0)	18	25.4	14	19.7	4	16.7
		*S. gallinarum*	8 (20.0)	13	18.3	9	12.7	4	16.7
		*S. lentus*	4 (10.0)	6	8.5	4	5.6	2	8.3
		*S. lugdunensis*	4 (10.0)	5	7	4	5.6	1	4.2
		*S. epidermidis*	5 (12.5)	5	7	2	2.8	3	12.5
		*S. chromogenes*	5 (12.5)	5	7	4	5.6	1	4.2
		*S. sciuri*	5 (12.5)	5	7	3	4.2	2	8.3
		*S. warneri*	3 (7.5)	3	4.2	2	2.8	1	4.2
		*S. simulans*	2 (5.0)	3	4.2	3	4.2	0	0
		*S. arlettae*	3 (7.5)	3	4.2	3	4.2	0	0
		*S. vitulinus*	2 (5.0)	2	2.8	2	2.8	0	0
		*S. auricularis*	1 (2.5)	1	1.4	1	1.4	0	0
		*S. schleiferi*	1 (2.5)	1	1.4	1	1.4	0	0
		*S. saprophyticus*	1 (2.5)	1	1.4	1	1.4	0	0
		*S. capitis*	1 (2.5)	1	1.4	0	0	1	4.2
		*S. carnosus*	1 (2.5)	1	1.4	1	1.4	0	0
	Others	*Aerococcus viridans*	1 (2.5)	1	1.4	1	1.4	0	0
		*Enterococcus faecium*	1 (2.5)	1	1.4	1	1.4	0	0
		*Leuconostoc cremoris*	1 (2.5)	1	1.4	1	1.4	0	0
		*Kocuria kristinae*	1 (2.5)	1	1.4	1	1.4	0	0
		*Alloiococcus otitis*	1 (2.5)	1	1.4	1	1.4	0	0

CPS—coagulase-positive staphylococci; CNS—coagulase-negative staphylococci.

**Table 3:** The generalized binary logistic mixed model evaluating the influence of SRLV infection on the prevalence of bacteria in milk samples controlled for possible confounders.

Variable	Regression Coefficient (SE)	Model Parameter	*p*-Value	OR (CI 95%)
Goat	0.00 (0.00)	-	0.999	-
Constant	0.69 (0.55)	-	-	-
Parity				
1st lactation	- ^a^	-	-	-
2nd lactation	−2.08 (0.96)	−2.16	0.032 *	0.13 (0.02, 0.84)
3rd lactation	−1.87 (0.79)	−2.36	0.019 *	0.16 (0.03, 0.74)
4th lactation	−1.51 (0.71)	−2.13	0.034 *	0.22 (0.05, 0.89)
≥5th lactation	−3.23 (1.13)	−2.85	0.005 *	0.04 (0.004, 0.37)
SRLV status in parity classes				
SRLV-SN in any lactation	- ^a^	-	-	-
SRLV-SP in the 1st lactation	0.32 (0.80)	0.40	0.692	1.37 (0.28, 6.64)
SRLV-SP in the 2nd lactation	0.98 (1.02)	0.96	0.338	2.66 (0.36, 19.90)
SRLV-SP in the 3rd lactation	−0.27 (0.80)	−0.34	0.735	0.76 (0.16, 3.67)
SRLV-SP in the 4th lactation	0.39 (0.58)	0.68	0.495	1.48 (0.48, 4.61)
SRLV-SP in ≥5th lactation	2.59 (1.02)	2.55	0.012 *	13.4 (1.80, 99.2)
**Breed** ^b^				
PWI ^c^	- ^a^	-	-	-
PFI ^d^	0.04 (0.34)	0.11	0.909	1.04 (0.54, 2.02)
**Milking (stage of lactation)** ^b^				
20th day	- ^a^	-	-	-
40th day	0.33 (0.45)	0.73	0.466	1.39 (0.57, 3.36)
60th day	0.82 (0.46)	1.78	0.076	2.26 (0.92, 5.57)
150th day	1.06 (0.45)	2.36	0.019 *	2.89 (1.19, 7.01)
210th day	0.01 (0.47)	0.01	0.989	1.01 (0.40, 2.54)

^a^ baseline category; ^b^ variables eliminated from the model in the backward stepwise procedure; ^c^ PWI—Polish White Improved; ^d^ PFI—Polish Fawn Improved; * statistically significant at α = 0.05.

## Data Availability

The datasets used and/or analyzed during the current study are available from the corresponding author on reasonable request.

## Supplementary Materials

The following are available online at https://www.mdpi.com/article/10.3390/ani11071851/s1, Table S1: Prevalence of bacteria in milk samples from SRLV-positive and SRLV-negative goats in subsequent lactations.
