# Supplementary material for: Does Small Ruminant Lentivirus Infection in Goats Predispose to Bacterial Infection of the Mammary Gland? A Preliminary Study
Source: Animals (Basel). 2021 Jun 22;11(7):1851. doi: 10.3390/ani11071851 (PMC8300097; doi:10.3390/ani11071851)
Supplement: Supplementary file 1 [file animals-11-01851-s001.zip › animals-1189015-supplementary.pdf]

Table S1. Prevalence of bacteria in milk samples from SRLV-positive and SRLV-negative goats in subsequent lactations

**Lactation**	**SRLV status**				**p-value of χ^2 test**
	**seropositive (n=155)**		**seronegative (n=80)**		
	n	% (CI 95%)	n	% (CI 95%)	
1st (n=30)	11 / 15	73.3 (48.0, 89.1)	10 / 15	66.7 (41.7, 84.8)	0.693
2nd (n=20)	4 / 10	40.0 (16.8, 68.7)	2 / 10	20.0 (5.7, 51.0)	0.329
3rd (n=38)	4 / 21	19.0 (7.7, 40.0)	4 / 17	23.5 (9.6, 47.3)	0.735
4th (n=56)	13 / 33	39.4 (24.7, 56.3)	7 / 23	30.4 (15.6, 50.9)	0.489
≥5th (n=91)	39 / 76	51.3 (40.3, 62.2)	1 / 15	6.7 (1.2, 29.8)	0.002 / 0.008[a]

[a] Bonferroni correction
